# Ubiquitin Carboxyl-Terminal Esterase LI *(UCHLI)* SI8Y Polymorphism in Patients with Cataracts

**DOI:** 10.3109/13816810.2010.544360

**Published:** 2011-01-25

**Authors:** Thiemo Rudolph, Annica Sjölander, Mona Seibt Palmér, Lennart Minthon, Anders Wallin, Niels Andreasen, Gunnar Tasa, Erkki Juronen, Kaj Blennow, Henrik Zetterberg, Madeleine Zetterberg

**Affiliations:** 1 Department of Clinical Neuroscience and Rehabilitation, Institute of Neuroscience and Physiology, Section of Ophthalmology, The Sahlgrenska Academy at the University of Gothenburg, Mölndal, Sweden; 2 Department of Psychiatry and Neurochemistry, Institute of Neuroscience and Physiology, The Sahlgrenska Academy at the University of Gothenburg, Mölndal, Sweden; 3 Department of Clinical Chemistry and Transfusion Medicine, Institute of Biomedicine, The Sahlgrenska Academy at the University of Gothenburg, Göteborg, Sweden; 4 Department of Clinical Sciences, Clinical Memory Research Unit, Lund University, Lund and Neuropsychiatric Clinic, Malmö University Hospital, Malmö, Sweden; 5 Department of Geriatric Medicine, Memory Clinic, Karolinska University Hospital, Huddinge, Stockholm, Sweden; 6 Department of Human Biology and Genetics, Institute of General and Molecular Pathology, University of Tartu, Tartu, Estonia

**Keywords:** cataract, uchll, ubiquitin

## Abstract

*Background:* Cataract is characterized by light-scattering protein aggregates. The ubiquitin-proteasome system has been proposed a role in proteolytic removal of these protein aggregates. Ubiquitin carboxyl-terminal esterase L1 (UCHL1) is a de-ubiquitinating enzyme with

important functions in recycling of ubiquitin. A protective role of the p.S18Y polymorphism of the *UCHL1* gene has been shown in Parkinson's disease. The current study aimed to examine possible effects on cataract formation.

*Methods:* Patients with cataract (n = 493) and controls (n = 142) were analyzed for the *UCHL1* p.S18Y polymorphism using dynamic allele-specific hybridization.

*Results:* Significant differences were observed in allele and genotype frequencies of the p.S18Y polymorphism between controls and cataract patients, where a positive *UCHL1* allele A carrier status was associated with the cataract diagnosis (adjusted OR 1.7 [95% CI = 1.1-2.6] p = 0.02). No significant differences were seen in genotype distribution when stratifying for type of cataract. Nor did the mean age at cataract surgery differ between genotypes.

*Conclusion:* The current study does not support a protective role for the *UCHL1* S18Y polymorphism in cataract development, but may instead suggest a disease-promoting effect.

## INTRODUCTION

Ubiquitin carboxyl-terminal esterase L1 (UCHL1), also known as ubiquitin carboxy-terminal hydrolase and neuron-specific protein gene product 9.5 (PGP 9.5), is one of the most abundant proteins in the brain and therefore used as a sensitive neural marker.^1^ It belongs to the family of de-ubiquitinating proteins (DUBs) and is as such a component of the ubiquitin-proteasome system (UPS). The major function of UCHL1 can be described as mono-ubiquitin recycling, helping to maintain monomeric ubiquitin levels. Interestingly, as a dimer it reverses its function and becomes an ubiquitin-protein ligase resembling the E3 enzymes of the UPS.^2^ The *UCHL1* gene gained special interest after it had been linked to a family with an autosomal dominant missense mutation (p.I93M) causing Parkinson's disease (PD).^3^ While this mutation seems to be very rare, another, more frequent *UCHL1* polymorphism has been discovered in the course of a search for p.I93M mutants. The p.S18Y variant (c.53C >A, rs id 5030732) shows diminished dimerization and ligase activity.^2^ It has been ascribed a protective role for PD by some authors,^4^–^6^ while other studies found no association with the disease.^7^ For other neurodegenerative diseases, the reports are similarly contradictive. In Huntington's disease, the *UCHL1* p.S18Y variant is linked to age at onset.^8^ For Alzheimer's disease (AD), a Chinese study has demonstrated lower frequencies of the A allele and the AA genotype in female AD patients as compared to female controls.^9^ However, other studies could not find an association between *UCHL1* genotypes and AD.^10^,^11^ UCHL1 is expressed mostly in neural tissues, but there are also some reports of other non-neural tissues expressing UCHL1.^12^ There are few reports on UCHL1 expression in the eye. In the retina, expression seems to be most abundant in the horizontal and ganglion cells.^13^ To our knowledge there is only one report on UCHL1 expression in the lens, showing increased expression in the lens epithelial cells of atopic cataracts.^14^ UPS activity is present in the aging lens and seems to diminish both with increasing turbidity and from the lens epithelium to the nucleus.^15^ Cataract is opacification of the eye lens resulting from the aggregation of lens proteins, mainly the so called crystallins. The UPS has been suggested to play a pivotal role in preventing the formation of such light-scattering protein aggregates by proteolytic degradation.^16^ The present study thus aimed to investigate if there is an association between the *UCHL1* p.S18Y polymorphism and cataract.

## PATIENTS AND METHODS

After informed consent, patients with senile cataract and control individuals were recruited from two ophthalmic clinics in the town of Tartu and the South-Estonian area. The study was approved by the Ethical Commission at the University of Tartu, Estonia. Prior to surgery, the type of cataract was determined using bio-microscopy and ophthalmoscopy. Secondary cataracts were excluded. The case group included 493 cataract patients (76 with nuclear, 154 with cortical, 116 with posterior subcapsular, and 147 with mixed opacities). The reported age is close to the time of surgery. For control subjects, only individuals above the age of 60 years were included, yielding 142 individuals with a mean age (±SD) of 68 ±5.3 (range 61 to 90 years). Mean age in the cataract group was 72 ±8.7 years (range 47-93 years).

The *UCHL1* (gene map locus 4p14; [Entrez gene ID: 7345]) p.S18Y, c.53C>A polymorphism (rs5030732) was analyzed using the Dynamic Allele Specific Amplification (DASH) tehnology as described earlier.^17^ The PCR were carried out with HotStarTaq DNA Polymerase® (QIAGEN, Hilden, Germany) in a final volume of 25 μl, containing 5-20 ng of template DNA. Optimal conditions were: 1 mM MgCl2,0.2 mM dNTPs, 0.02 U Taq polymerase, 0.16 pmol/μl of the forward biotinylated primer (5‘Biotin-GCCGCCTT-GTCTCCTCTCAGCAG3’) and 0.78 pmol/μl of the reverse primer (5‘GTCACTGGCCTGCGACCCC3’), (Invitrogen, Paisley, UK) in 1 x PCR buffer (Roche, Mannheim, Germany). The cycling profile was: 15min at 95 °C, then 39 cycles: 30 sec at 95°C, 45 sec at 60°C, 45 sec at 72°C and finally 10min at 72 °C. To identify *UCHL1* alleles the probe 5‘GACAGAAACGCACTTGT-Rox3’ (MWG Biotech, London, United Kingdom) was used. The accuracy of the DASH method was verified by DNA sequencing of 15 individuals representing the different *UCHL1* genotypes (five of each genotype).

Primary analyses compared differences between the cataract patients and control subjects regarding age, sex, genotype and allele frequencies using Student's t-test, Fisher's exact test and Pearson's chi-square test. Secondary analysis of *UCHL-1* allele A-carrier status was performed with a binary logistic regression model with diagnosis (cataract versus control) as dependent variable and allele positivity, age and sex as independent variables. Significance was set at P<0.05. SPSS 17.0 (SPSS Inc., Chicago, Il) was used as statistic software.

## RESULTS

Mean age and sex distribution for the cataract and control group is shown along with genotype distribution, allele frequency and *UCHL1* allele A-carrier status in Table 1. The allele frequencies found in this study were in accordance with previous reports on caucasian populations, including neighbouring countries (see Swedish study by Belin et al.).^4^,^6^ The genotype distribution for both cataract and control group were in Hardy-Weinberg equilibrium. Significant differences in genotype and allele distributions were seen between the control group and the cataract patients. A positive *UCHL1* allele A-carrier status was significantly higher among cataract patients as compared to the control group (p = 0.015). This association remained after correcting for age and sex, using a binary logistic model (adjusted OR 1.7 [95% CI = 1.1-2.6] p = 0.02), see Table 2. Stratification of the data according to type of cataract showed a consistently higher percentage of both homozygotes and heterozygotes for the *UCHL1* allele A as compared to controls. This overrepresentation was statistically significant for posterior subcapsular and mixed cataract only (Table 3). Age at surgery did not vary significantly between *UCHL1* p.S18Y genotypes, as evident in Table 4.

**TABLE 1:** Demographic and genetic characteristics of patients with cataract and controls.

	Control	Cataract	p-value
Number (total)	142	493	
Age (years), mean±SD	68 ± 5.3	72 ± 8.7	<0.001*
Sex female/male, n (%)	101/41 (71.1/28.9)	342/151 (69.4/30.6)	0.39†
UCHL1			
Genotype frequencies, n (%)			
AA	3 (2.1)	20 (4.1)	0.039‡
AC	31 (21.8)	153 (31.0)	
CC	108 (76.1)	320 (64.9)	
Allele frequency, n (%)			
A	N = 37 (13.0)	N = 193 (19.6)	0.011†
C	N = 247 (87.0)	N = 793 (80.4)	
Allele A-carrier, n (%)	N = 37 (23.90)	N = 193 (35.10)	0.015†

* Student's t-test

† Fisher's exact test

‡ Chi-2 test, Pearson's

**TABLE 2:** Binary logistic regression of cataract versus control group for age, sex and *UCHL1* allele A-carrier status.

	B	S.E.	adjusted OR	95% C.I.	p-value
Age	0.06	0.01	1.06	1.03-1.08	<0.001
female gender	-0.17	0.22	0.84	0.55-1.29	0.43
UCHL1, allele A-carrier	0.52	0.22	1.68	1.09-2.59	0.02

B = Regression coefficient, S.E. = Standard error, OR = odds ratio, CI = Confidence interval

**TABLE 3:** Cataract type versus *UCHL1* genotype.

		Cataract type			
*UCHL1* genotype	Controls	Cortical	nuclear	post. subcapsular*	mixed*
p-value		0,36	0,16	0,036	0,040
N (total)	142	154	76	116	147
AA	3	4	3	8	5
	2,1%	2,6%	3,9%	6,9%	3,4%
CA	31	44	24	35	50
	21,8%	28,6%	31,6%	30,2%	34,0%
CC	108	106	49	73	92
	76,1%	68,8%	64,5%	62,9%	62,6%

* p<0.05, Fisher's exact test, as compared to control group

**TABLE 4:** *UCHL1* S18Y genotypes vs age at surgery in the cataract group.

*UCHL1* genotype		
AA	N	20
	**Mean (years)**	**72,3**
	Median (years)	72,5
	SD (years)	11,7
CA	N	153
	**Mean (years)**	**72,2**
	Median (years)	71,0
	SD (years)	8,5
CC	N	320
	**Mean (years)**	**71,9**
	Median (years)	72,0
	SD (years)	8,7

## DISCUSSION

It has been shown that with ageing of the lens, tissue-specific crystallin proteins are subjected to a large number of posttranslational changes, including oxidative modifications.^18^

The resulting conformational changes cause protein aggregation and subsequent light scatter, hence cataract.^19^ In mammalian cells, including lens cells/lens fibers, the ubiquitin-proteasome system (UPS) is the major proteolytic system for degradation of aberrant proteins. It has been shown that mildly oxidized crystallin proteins are better substrates for the UPP than native proteins, supporting a role for the UPS in clearing the lens from defect proteins.^16^

Aggregation of aberrant proteins is also a hallmark of several neurodegenerative diseases, including Alzheimer's (AD), Huntington's (HD), and Parkinson's (PD) diseases. Accordingly, the UPS has been suggested to play a major role in preventing the formation of pathological protein aggregates in these diseases as well.^20^

A number of reports have demonstrated a protective effect of the *UCHL1* p.S18Y polymorphism against sporadic PD in different populations,^4^,^6^,^21^,^22^ although conflicting data exist.^23^ As for AD, data on *UCHL1* genotype frequencies and its effect on risk of AD is scarce and conflicting.^9^,^10^ To our knowledge, there are no previous reports on the effect of the *UCHL1* p.S18Y genotype distribution in cataract patients.

Cataract is a strongly age-dependent disorder; in the Beaver Dam Eye Study, the 5-year cumulative incidence of nuclear cataract increased from 2.9% in persons aged 43 to 54 years at baseline to 40% in those aged 75 or older.^24^ In this study, the reported age for the cataract patients corresponds to the year of surgery. While it is not self-evident that this age corresponds to the age at onset, we find it reasonable to assume that the two variables correlate, at least at the group level. For an age-dependent disease, one can assume that carriers of a protective gene variant should on average have a higher age at onset. However, when stratifying the cataract group according to *UCHL1* p.S18Y genotype, there was no significant difference in mean age, neither indicating a protective nor disease-promoting effect. In contrast, the *UCHL1* allele A frequency was significantly higher in the cataract group compared to the control group. After stratifying for clinically different types of cataract, significance was lost for nuclear and cortical cataract. If this is a random effect due to sample size or if this reflects pathophysiological differences between cataract subgroups cannot be safely concluded from our data. Nevertheless, these findings may suggest a possible disease-promoting effect and argue against a protective effect of the *UCHL1* p.S18Y polymorphism in cataract. However, the associations found were quite weak and argue against any pivotal role for the *UCHL1* p.S18Y polymorphism in cataract disease. In addition, a limitation of the present study is that multiple loci or several diseases were not tested.

Given the complexity of the UPS and its essential functions in all organ systems, it is not surprising that it has been implicated in the pathophysiology of a large number of both congenital and acquired diseases and in the process of ageing itself.^25^,^26^ Several polymorphisms of the UPS and associated proteins have been described, the majority of which are disease-promoting.^27^,^28^ On the contrary, the p.S18Y polymorphism of the *UCHL1* gene appears to confer at least a moderate protection in Parkinson's disease.^29^

In this study, the p.S18Y variant was underrepre-sented in the control group, hence excluding a protective role of this *UCHL1* polymorphism in cataract. Considering the multiple functions of UCHL1 with regard to the UPS, such as recycling of ubiquitin, the processing of ubiquitin precursors and ubiquitin ligase activity,^30^ it is possible that separate functions of the enzyme have opposed effects in the pathogenesis of different diseases. In neurodegenerative disorders characterized by protein aggregation, recent studies have suggested that it is not the protein aggregates in themselves that lead to neuronal death, but rather the preceding oligomers that are toxic to the neurons.^31^ Similar toxic effects could also be damaging to the lens, especially in the more metabolically active parts such as the epithelium and the superficial lens fibers. In the metabolically quiescent lens nucleus however, light-scattering effects of large aggregates of lens proteins should be a more important pathogenetic feature than in the outer lens regions and also as compared to neuronal tissue.
